# Virtual parental presence with coaching for reducing preoperative anxiety in children: a feasibility and pilot randomized controlled trial

**DOI:** 10.1016/j.bjane.2024.844533

**Published:** 2024-06-26

**Authors:** Clyde T. Matava, Martina Bordini, Ben O’ Sullivan, Gabriela Alcaraz Garcia-Tejedor, Nan Gai, Guy Petroz, Conor Mc Donnell, Fahad Alam, Katie Brazel, Monica Caldeira-Kulbakas

**Affiliations:** a The Hospital for Sick Children, Department of Anesthesia, Toronto, Ontario, Canada; b University of Toronto, Department of Anesthesiology and Pain Medicine, Toronto, Ontario, Canada; c University of Bologna, Department of Medical and Surgical Sciences (DIMEC), Bologna, Italy; d Hospital for Sick Children, Child Life Department, Toronto, Ontario, Canada; e Sunnybrook Health Sciences Centre, Department of Anesthesia, Toronto, Ontario, Canada

**Keywords:** Anxiety, Pediatric Anesthesia, Videoconference, Parental Presence at Induciton of Anesthesia, Preoperative anxiety, MYPAS, PHBQ

## Abstract

**Background:**

Preoperative anxiety in children causes negative postoperative outcomes. Parental presence at induction is a non-pharmacological strategy for relieving anxiety; nevertheless, it is not always possible or effective, namely when parents are overly anxious. Parental presence via video has been demonstrated to be useful in other contexts (divorce, criminal court). This study reports the feasibility of a randomized controlled trial to investigate the effect of video parental presence and parental coaching at induction on preoperative anxiety.

**Methods:**

The study was a randomized, 2 × 2 factorial design trial examining parental presence (virtual vs. physical) and coaching (provided vs. not provided). Feasibility was assessed by enrollment rate, attrition rate, compliance, and staff satisfaction with virtual method with the NASA-Task Load Index (NASA-TLX) and System Usability Scale (SUS). For the children's anxiety and postoperative outcomes, the modified Yale Preoperative Anxiety Scale (mYPAS) and Post-Hospitalization Behavioral Questionnaire (PHBQ) were used. Parental anxiety was evaluated with the State-Trait Anxiety Inventory (STAI) questionnaire.

**Results:**

A total of 41 parent/patient dyads were recruited. The enrollment rate was 32.2%, the attrition rate 25.5%. Compliance was 87.8% for parents and 85% for staff. The SUS was 67.5/100 and 63.5/100 and NASA-TLX was 29.2 (21.5–36.8) and 27.6 (8.2–3.7) for the anesthesiologists and induction nurses, respectively. No statistically significant difference was found in mYPAS, PHBQ and STAI.

**Conclusion:**

A randomized controlled trial to explore virtual parental presence effect on preoperative anxiety is feasible. Further studies are needed to investigate its role and the role of parent coaching in reducing preoperative anxiety.

## Introduction

Preoperative anxiety in children negatively affects postoperative outcomes and parents’ and children's experience of the operating room. However, up to 50% of children develop behavioral stress and anxiety before surgery.^1^ Consequences of preoperative anxiety include behavioral changes (separation anxiety, sleep disturbance, and temper tantrums)^2^ and more painful postoperative recovery,^3^ with correlation between anxiety levels and postoperative pain levels.^4^

Multiple pharmacological and non-pharmacological prevention strategies have been explored.^5^ Among non-pharmacological methods, Parental Presence at Induction of Anesthesia (PPIA) has been shown to reduce parent and patient anxiety without impeding operating room efficiency.^6^ However, a recent cross-sectional survey reported a rate of PPIA in a tertiary children hospital of 16.9%;^7^ among the reasons for not accompanying their child in the operating room, parental anxiety was reported by 14% of parents. It is known that the presence of an overly anxious parent has no obvious benefit for the child during induction.^8^ Previous studies^9^ failed to demonstrate that preparation of parents before PPIA reduces preoperative anxiety.

Another non-pharmacological intervention is the use of technology as cartoon/video distraction technology. In a Randomized Control Trial (RCT), video distraction showed similar effects on preoperative anxiety compared to PPIA.^10^ Technology may be helpful in situations in which PPIA may not be useful, such as for parents anxious in the operating room environment or limited access of family to patient care settings (e.g., as in the COVID-19 pandemic).

To date, there is a paucity of data assessing the role of PPIA via video; however, early work in this area highlighted that infants and toddlers who saw their parents on video during divorce, incarceration, or criminal court proceedings demonstrated the same behavioral and play patterns as though parents were physically present,^11^ therefore it may follow that children could also benefit from virtual Parental Presence at Induction of Anesthesia (vPPIA) in healthcare settings. This study aimed to investigate the effects of virtual parental presence and parental coaching on children's level of anxiety at induction of anesthesia, with the hypothesis of a synergistic effect of virtual presence and parental coaching in reducing parental anxiety. As part of the response to the COVID pandemic, our institution among others halted all clinical studies, especially those requiring the use of personal protective equipment.^12^, ^13^, ^14^ We therefore report the study as a pilot and feasibility trial.

## Methods

### Ethics

The study received approval as an RCT at the Hospital for Sick Children, Toronto (REB# 1000053821). The study was registered on the clinicaltrials.gov platform (ID NCT02950415). Written informed consent was obtained from parents/guardians. Staff consent was implied via completion of the questionnaires.

### Study design

The study was a pilot randomized, 2 × 2 factorial design trial examining parental presence (virtual vs. physical) and coaching (provided vs. not provided). Specifically, patient/parent dyads were randomized to one of four groups: virtual Parental Presence with coaching (vPPIA+), virtual Parental Presence without coaching (vPPIA-), physical Parental Presence with coaching (pPPIA+), and Physical Parental presence without coaching (pPPIA-).

### Sample size

The primary objective of this report is feasibility. Our available sample size of 41 patient/parent dyads is not powered to detect statistically significant differences among groups. The original RCT was planned to achieve adequate statistical power. Assuming a two-tailed alpha = 0.05, beta = 0.2 (power = 80%), and a dropout rate of 20%, 80 patient/parent dyads in each group should have been recruited, for a total of 320 patient/parent dyads.

### Participants

The study was conducted from September 2017 to August 2019 in the operating rooms of The Hospital for Sick Children, Toronto. Children aged from 18 months to 12 years who were undergoing ambulatory day-case surgery, with American Society of Anesthesiologists (ASA) physical status I, II, III were eligible for inclusion. Patient/parent dyads who agreed to participate were randomly allocated to one of the study groups. All patient/parent dyads enrolled received treatment as per group allocation. A complete list of exclusion criteria and changes made to the protocol are provided in the Supplementary material - Appendix A.

### Randomization

Group assignments were kept in sealed, opaque, sequentially numbered envelopes and opened the morning of surgery by a designated research assistant who was not involved in data collection. Participants were assigned to a group only after baseline assessments were completed to prevent group selection bias.

### Interventions

#### vPPIA groups

Facetime™ video platform designed by Apple Inc was used on two iPads™. FaceTime is a peer-to-peer videotelephony available on devices that run iOS and Macintosh computers.

Patients in the vPPIA groups were brought to the operating room while parents were accompanied to an anteroom where they could see their child via video. Patients were directed by the research assistant to focus on the video monitor in the operating room while induction took place. The video link remained live until the patient was asleep and the anesthesiologist informed the parent that the child was unaware. A research assistant remained with the parent throughout the induction to help with the technology.

#### pPPIA groups

Patients were accompanied by the parent into the operating room and remained until patient loss of awareness; the parent was then accompanied back to the waiting room by a research assistant.

#### Coaching

The coaching sessions occurred with a Child Life Specialist at SickKids. Parents allocated to the coaching groups were shown a brief video that outlined the behaviors they should demonstrate and those less desirable during induction.

Participants were presented a standardized video. For the vPPIA+ group emphasis was placed on the use of words to support their child, for the pPPIA+ group emphasis was placed on both words and actions to support their child.

Parents were also provided with a handout (Supplementary material - Appendix B) summarizing the desired and undesired behavior. Parents in the PPIA- groups (both physical and virtual) did not receive coaching or handouts.

### Anesthesia induction

After monitoring, mask induction was conducted using a sevoflurane, nitrous oxide, and oxygen mixture. Patients were encouraged to lie down during induction but could remain sitting up if needed. Anesthesia would proceed with inhalational agents for induction.

### Outcomes, instruments and measurements

#### Primary outcome – Feasibility

To assess feasibility, enrollment (number of enrolled subjects/number of eligible subjects) and attrition rate (number of participants that withdraw/number of participants randomized), compliance among participants in completing study materials and satisfaction were evaluated.

#### Secondary outcomes

##### Anxiety in children at induction of anesthesia and induction compliance

The modified Yale Preoperative Anxiety Scale (mYPAS)^15^ was used, which was developed specifically to measure anxiety at anesthesia induction. The scale looks at activity, facial expression, alertness and arousal, vocalization, and interaction with adults. It has a good validity against the State-Trait Anxiety Inventory for Children, as well as good intra- and inter-observer reliability.^16^ Scores range from 22.5 to 100, with higher scores indicating greater anxiety. The Induction Compliance Checklist (ICC) is an observational scale that describes the compliance of a child during induction.^17^ This scale is used routinely at our institution. A higher ICC score corresponds to lower compliance at induction.

##### Child temperament

The Emotionality Activity Sociability Impulsivity Instrument of Child Temperament (EASI) is a parent-reported instrument that assesses four temperament categories in children. The tool has good validity and test-retest reliability.^18^ Scores range from 5 to 25 for each category, with higher scores denoting higher baseline emotionality, activity, sociability, or impulsivity. Higher emotionality has been linked to increased preoperative anxiety, whereas highest activity with reduced anxiety.^19^

##### Posthospital negative behaviors

The Posthospitalization Behavior Questionnaire (PHBQ) was utilized for child postoperative assessment. This tool is a questionnaire for parents that examines the child's general anxiety, separation anxiety, sleep anxiety, eating disturbance, aggression towards authority, and apathy and withdrawal. Higher scores indicate negative behavior change. Internal consistency is reported to be adequate for subscale scores and excellent for overall scores.^20^

##### Parental anxiety at induction of anesthesia

At baseline and postoperatively, the State-Trait Anxiety Inventory (STAI) was used for parental anxiety. STAI is the gold standard for assessing anxiety in adults.^21^ It comprises 20 questions and has good test-retest reliability. Total scores range from 20 to 80, with higher scores indicating higher levels of anxiety.

##### Satisfaction

A standard satisfaction questionnaire used routinely at our institution was employed to measure parental satisfaction. Staff satisfaction was measured using the NASA Task Load Index (NASA-TLX) and the System Usability Scale (SUS). The NASA-TLX evaluates the workload of an activity; the workload is considered low for a score of 0–9, medium for 10–29, somewhat high for 30–49, high for 50–79 and very high for 80–100. To evaluate acceptability and usability the System Usability Scale (SUS) was used: a score of > 70 is regarded as acceptable; a score of at least 71.4 suggests good usability, while a score of 85.5 suggests excellent usability. The NASA-TLX is reported to have good validity and reliability.^22^ while the SUS is reported to have good validity and excellent reliability.^23^

### Study flow

Data collection took place across three timepoints: pre-anesthesia (baseline), during induction of anesthesia, and postoperatively. A flow chart demonstrating the timepoints and data to be collected during each period is provided in the Supplementary material -Appendix C.

#### Baseline assessments

Following consent, the mYPAS was used to assess the child's baseline anxiety before surgery, at operating room entrance, by two research assistants assigned to the patient. Following each assessment, the research assistants met to reach consensus on the final score. All assessors underwent standardized training to achieve an inter- and intra-rater reliability of at least 95% for the mYPAS. The parent was self-administered STAI and EASI. All parents were provided with verbal anesthesia induction preparatory information focusing on four areas: operating room environment, parent's role during induction, what to expect as the child is induced, and post-induction procedures.

#### Child anxiety and cooperation assessment during induction of anesthesia

Patient's anxiety level was measured again using the mYPAS at the time of induction by the two research assistants assigned to the patient. Following each assessment, the research assistants met to reach consensus on the final score. The child's cooperation at induction was recorded using the ICC by one of the research assistants assigned to the patient.

#### Postoperative assessments

Immediately following surgery, the anesthesiologist and the induction nurse were asked by one of the research assistants assigned to the patient to complete the NASA-TLX and the SUS. Two-three days after surgery, parents were contacted by the research assistant assigned to them by phone to complete the PHBQ.

### Statistical analysis

Data were summarized as frequencies or percentages for categorical variables and mean for continuous variables, reported with a 95% Confidence Interval (mean, [95% CI]). The Chi-square test was used to compare categorical variables and student's t test was used to compare two continuous variables. Analysis of variance (ANOVA) was also conducted to compare the primary outcome among groups. The two-tailed *p*-value ≤ 0.05 was considered statistically significant. Statistical analyses were performed using SAS (version 9.4; SAS Institute Inc., Cary, NC, USA).

## Results

### Participants

A total of 41 parent/patient dyads participated in the study. The demographic information of the participants is shown in Table 1. The mean age of patients was 7.5 years (5.2–9.7). Overall, 18 parents (43.9%) had previous experience with anesthesia for their child. No significant differences were observed among the groups in terms of age, sex, parents’ previous experience, and number of previous anesthetics; EASI score and STAI preoperatively were not statistically significantly different among groups (Table 2).

**Table 1:** Participants demographics score.

	Virtual Parental Presence with Coaching (vPPIA+)	Virtual Parental Presence without Coaching (vPPIA-)	Physical Parental Presence with Coaching (pPPIA+)	Physical Parental Presence without Coaching (pPPIA-)
**Age: mean [95% CI]**	7.5 [5‒9.9]	7.7 [5.4–10]	9 [7.2–10.8]	5.6 [4.1–7.1]
**Sex: n (%)**				
Female	7 (70%)	5 (50%)	3 (27.3%)	2 (20%)
Male	3 (30%)	5 (50%)	8 (72.7%)	8 (80%)
Total	10	10	11	10
**Previous experiences – Parent, n (%)**				
Yes	4 (40%)	3 (30%)	5 (45.5%)	6 (60%)
No	6 (60%)	7 (70%)	5 (45.5%)	4 (40%)
missing	0	0	1 (9%)	0
**Previous experiences – Patient, n (%)**				
0	9 (90%)	5 (50%)	9 (82%)	6 (60%)
1	0	1 (10%)	1 (9%)	3 (30%)
2	0	4 (40%)	0	0
> 2	1 (1%)	0	0	1 (10%)
Missing	0	0	1 (9%)	0

Data are expressed as absolute number (n) and percentage (%) and mean and 95% Confidence Interval [95% CI].

**Table 2:** Baseline assessment for parents (STAI) and children (EASI).

	Virtual Parental Presence with Coaching (vPPIA+)	Virtual Parental Presence without Coaching (vPPIA-)	Physical Parental Presence with Coaching (pPPIA+)	Physical Parental Presence without Coaching (pPPIA-)
**STAI pre-op**	39.3 [29.9–48.7]	33.6 [26.3‒40.9]	35.6 [28.2–42.9]	34.8 [28.4–0.68]
**EASI**				
Emotionality	13.6 [12‒15.2]	14 [11.3–16.7]	12.7 [10.8–14.6]	16 [14.4‒17.6]
Activity	17.4 [14.4‒28.4]	15.6 [13.4–17.8]	13.5 [10.8‒16.2]	18.2 [16.5‒19.9]
Sociability	17.3 [14.8–19.8]	16.8 [15.5‒18.4]	16.8 [14.7‒18.9]	17.5 [16.6‒18.4]
Impulsivity	11.8 [9.9‒13.7]	10.5 [8.3‒12.7]	10.4 [8.7‒12.2]	11.6 [9.2‒14.4]

Data are expressed as mean (n) and 95% Confidence Interval [95% CI].

### Feasibility

#### Enrollment rate and attrition

**Figure 1:**
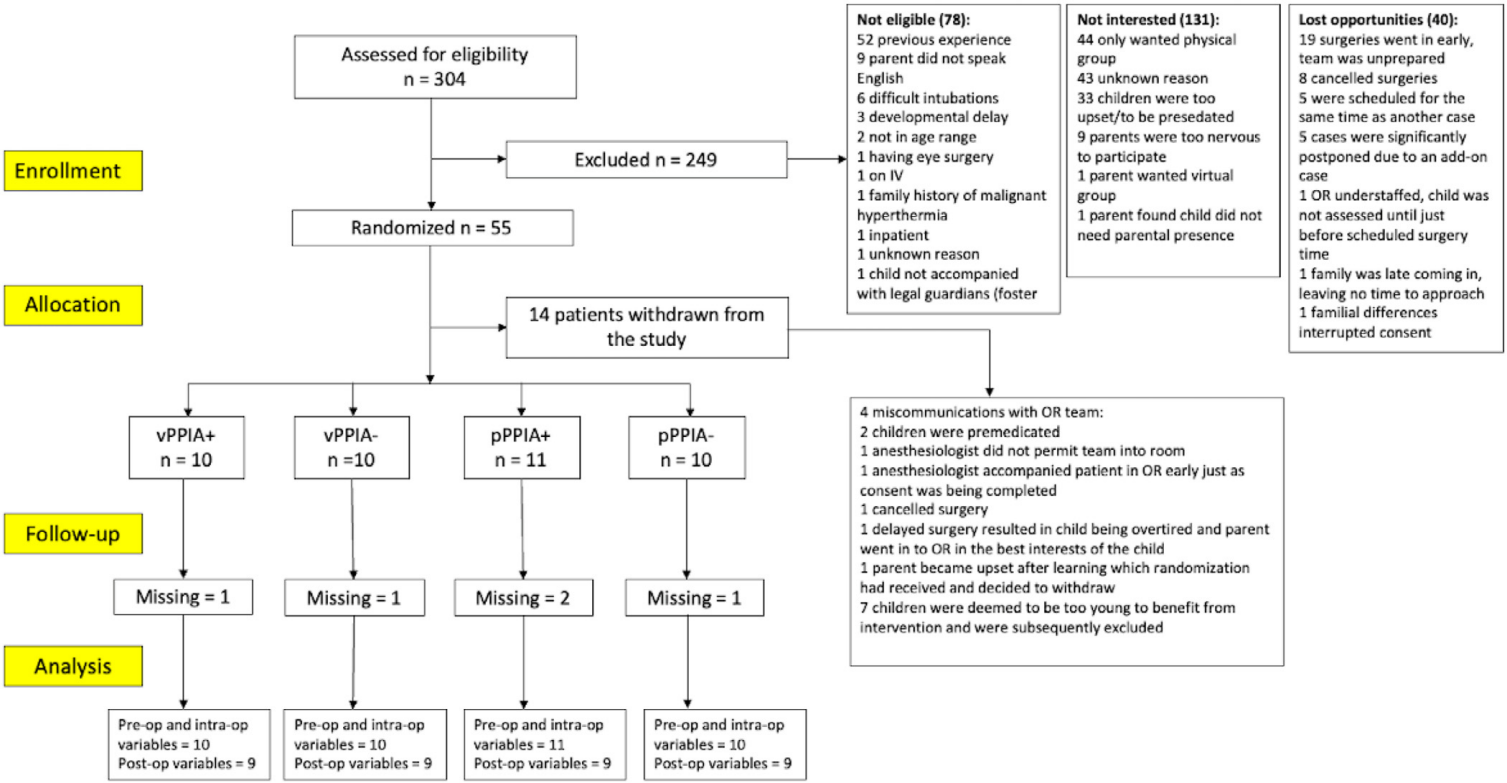
CONSORT 2010 diagram of the study flow and reasons of participants not eligible/excluded.

In total, 304 parents were approached for eligibility and assessment, 171 being eligible. Fifty-five participants consented to the study (enrollment rate 32.2%). Among the 249 not eligible/excluded, 52.6% of parents were not interested in the study, 30.9% were not eligible, with reasons of exclusion shown in Figure 1. After randomization, 14 participants withdrew from the study. The overall attrition rate was 14/55 (25.5%). The remaining participants were equally distributed among groups (10 vPPIA+, 10 vPPIA-, 11 pPPIA+, 10 pPPIA-) and there were no statistically significant differences on the baseline variables assessed. Figure 1 shows the flow of participants through the study following the CONSORT statement.^24^

#### Compliance

Among parents, 36/41 completed all the study tasks (compliance of 87.8%); the uncompleted tasks regarded the postoperative assessments (namely, parental satisfaction questionnaire). For the OR staff, compliance for completion of the SUS and NASA-TLX was 18/20 (90%) and 16/20 (80%), for anesthesiologists and induction nurses, respectively.

#### Satisfaction

Parental satisfaction was rated as “excellent” in more than 50% of parents, overall, but no statistically significant difference was observed among the groups. Parents’ outcomes are shown in Table 3.

**Table 3:** Parental post-operative anxiety and parental satisfaction.

	Virtual Parental Presence with Coaching (vPPIA+)	Virtual Parental Presence without Coaching (vPPIA-)	Physical Parental Presence with Coaching (pPPIA+)	Physical Parental Presence without Coaching (pPPIA-)	*p-*value
**STAI post-op**	39.7 [32.7‒46.7]	41.4 [34.7‒48.1]	38 [30.9–45]	36.5 [29.7‒43.3]	0.71
**Parental satisfaction**					0.63
Excellent	7 (77.8%)	6 (66.7%)	6 (66.7%)	5(55.6%)	
Very Good	2 (22.2%)	3 (33.3%)	2 (22.2%)	4(44.4%)	
Good	0	0	1 (11.1%)	0	
Total	9	9	9	9	
Missing	1	1	2	1	

Data are expressed as absolute number (n) and percentage (%) and mean and 95% Confidence Interval [95% CI].

For anesthesiologists and induction nurses, the SUS-score for video system usability was 67.5 (59.5–77.5) and 63.5 (53.5–73.6) out of 100, respectively. The NASA-TLX Score was 29.2 (21.5–36.8) for the anesthesiologists and 27.6 (8.2–37) for the induction nurses (Supplementary material - Appendix D). The SUS and NASA-TLX scores for the groups vPPIA+ and vPPIA- are provided in Table 4.

**Table 4:** Satisfaction of anesthesiologists and induction nurses.

	Virtual Parental Presence with Coaching (vPPIA+)	Virtual Parental Presence without Coaching (vPPIA-)	Virtual Parental Presence (both coaching and without-coaching)
**SUS-AN**	73.7 [66‒81.5]	61.2 (46.6‒75.9)	67.5 (59.4‒75.6)
**SUS-IN**	68.5 [53.5‒83.5]	58.1 [42.3‒74]	63.5 [53.5‒73.6]
**NASA TX-AN**	24.3 [12.1‒36.6]	34 [23.5‒44.5]	29.2 [21.5‒36.8]
**NASA TX-IN**	16.9 [9.7‒24.2]	39.5 [23.4‒55.7]	27.6 [18.2‒37.1]

Data are expressed as absolute number (n) and percentage (%) and mean and 95% Confidence Interval [95% CI].

### Secondary outcomes

#### Child and parent anxiety

The study was not powered to assess statistical significance, however statistical analysis was employed to look at results. The mean baseline mYPAS score in the vPPIA+, vPPIA-, pPPIA+, and pPPIA- were 27.7 (22.3–33), 26.7 (23–30.3), 25.6 (23.3–27.9), and 23.8 (22.7–25), respectively (*p* = 0.35). The lowest mYPAS score at induction was observed in the vPPIA+ group. A non-statistically significant difference (*p* = 0.052) was observed in the mean difference between baseline-mYPAS and induction-mYPAS of the groups. The only intervention that decreased mYPAS score from baseline to induction was the vPPIA+ with a mean difference of -2.3 (-6.8–2.2). There was no statistically significant difference in the mean PHBQ scores (*p* = 0.14). The highest mean PHBQ score was reported for the pPPIA- group, 23.7 (11.9–35.4); the lowest mean PHBQ score was reported for the vPPIA- group (8.4 [-1.8–18.6]). An additional statistical analysis comparing vPPIA and pPPIA (with or without coaching) was conducted. A lower PHBQ score was observed for the vPPIA group, but not significantly different (*p* = 0.16). Postoperatively, STAI scores did not differ among groups (*p* = 0.71). Results are summarized in Table 5.

**Table 5:** Assessment of children anxiety at induction (mYPAS and ICC) and PHBQ score.

	Virtual Parental Presence with Coaching (vPPIA+)	Virtual Parental Presence without Coaching (vPPIA-)	Physical Parental Presence with Coaching (pPPIA+)	Physical Parental Presence without Coaching (pPPIA-)	*p-*value
**mYPAS (Baseline)**	27.7 [22.3–33]	26.7 [23–30.3]	25.6 [23.3–27.9]	23.8 [22.7‒25]	0.35
**mYPAS (Induction)**	25.3 [23.5‒27.1]	38.2 [22‒54.3]	27.9 [24‒31.8]	38.2 [26.2‒50.1]	0.1
**Mean Differences MYPAS**	-2.33 [‒6.8‒2.2]	11.5 [-4.8‒27.8]	2.3 [-3.2‒7.7]	14.3 [2.7‒26]	0.052
**ICC**	0.3 [0.2‒0.8]	1.5 [0.6‒3.6]	0 [0‒0]	0.6 [0.3‒1.5]	0.19
**PHBQ**	19.2 [6.4‒32]	8.4 [-1.8‒18.6]	23.3 [12.5‒34.1]	23.7 [12‒35.4]	0.14

Data are expressed as mean (n) and 95% Confidence Interval [95% CI].

## Discussion

We report the feasibility of a randomized, 2 × 2 factorial design trial, examining parental presence (virtual vs. physical) and coaching (provided vs. not provided). Overall, the study appears feasible, but the results highlight some considerations. First, an enrollment rate of 32.2% was obtained. Participant recruitment is of vital importance for adequately conducting a trial and it is known that often the accrual period is underestimated in the building phase of the trial.^25^ The obtained enrollment rate is to be considered in the allocation of sufficient time for participant recruitment. Second, a 25.5% attrition rate was reported. A review by Hewitt et al.^26^ suggests that loss to follow-up occurs in many trials – a loss of 20% or greater means that the presence of biases cannot be excluded.^27^ However, in our study, loss happened mainly at the randomization period and the subsequent treatment and control groups were evenly distributed. In a future trial, attention should be given to the attrition rate and where losses happen during the study flow. In our study, seven children were excluded after randomization because they were deemed too young to benefit from the intervention; raising the lower age limit for enrollment from 18 months to 24 months could be an effective change to decrease attrition. Most importantly, the trial was designed in a period when video interactions between parents and caregivers were uncommon. The COVID pandemic changed this and may perhaps have made it easier to recruit participants in the video arm.

The highest SUS score and lowest NASA-TLX score were obtained in the vPPIA+ group. This could be explained by the novelty of the technology at the time of the study. With increased use of videocalls in recent years clinicians and parents may be more adept with these technologies.^28^ It is reasonable to expect better SUS and NASA-TLX scores nowadays, due to technology improvement and higher use of videocall technology by people.

Mean mYPAS scores at induction were reduced in the vPPIA+ group. We hypothesized a synergic effect of parental coaching and video presence could improve preoperative anxiety. The theorical framework of this assumption is the modularity concept. Modularity has been defined as the partial representation of a person instead of the whole person, that ends up being accepted, based on activity.^29^ In this context, parental presence by video may enable modularity, resulting in reduced anxiety, primarily in children and secondarily in parents. Technology, supporting telepresence interactions through video, may enhance the concept of modularity as children may engage positively with a parent that is partially represented. Similarly, parents may also exhibit more positive and soothing communication with the child due to modularity.

It is known that pPPIA is not effective for parents who are anxious^8^ as they can transmit anxiety to the child, and that a consistent proportion of parents feel anxious in accompanying their child to the OR.^7^ In contrast, virtual PPIA may reduce parental anxiety, by omitting the parent's physical presence in the operating room, resulting in less or no transmission of anxiety to the child and this may be influenced by the source of the parent's anxiety. At the same time, parents who experience high anxiety while physically present in the OR can still provide comfort to their child via video. Hypothetically, virtual PPIA may also result in less performance anxiety and the subsequent highest satisfaction of OR staff. Other potential benefits of vPPIA may include the reduced need for extra-personnel to accompany and manage parents in the OR during induction and afterwards. An adequately powered study is needed to confirm all these potential advantages.

Coaching is another relatively low-cost intervention that could have an impact on preoperative anxiety. Video or paper tools could be provided to parents/guardian before surgery. In children, a recent RCT by Batuman et al.^30^ has shown a reduction in mYPAS and PHBQ scores in children exposed to an informational video before anesthesia and surgery. Additional research is needed to better delineate effects of coaching on parents and indirectly on their child.

### Limitations

The pilot results do not provide evidence of any benefits of vPPIA and coaching on preoperative anxiety. Since the feasibility results come from a single-center experience, they may not be applicable to other institutions. Given that the study was carried out before the COVID-19 pandemic, the enrollment rate and satisfaction might differ if the study were conducted today.

## Conclusion

The theoretical background of virtual parental presence is promising in reducing preoperative anxiety and a RCT to explore its effect on preoperative anxiety is feasible. Further studies are needed to investigate its role and the role of parental coaching in reducing preoperative anxiety.

## Ethical approval

This study received institutional ethics approval. Written informed consent was obtained from parents/guardians of the patients. Anesthesiologist and induction nurse consent was implied via completion of the NASA-TLX and SUS questionnaires.

## Data availability statement

The data that support the findings of this study are available from the corresponding author upon reasonable request.

## Declaration of competing interest

The authors declare no conflicts of interest.

## Appendix Supplementary materials

Image, application 1

Image, application 2

## Footnotes

Supplementary material associated with this article can be found in the online version at doi:10.1016/j.bjane.2024.844533.
