# Supplementary material for: Virtual parental presence with coaching for reducing preoperative anxiety in children: a feasibility and pilot randomized controlled trial
Source: Braz J Anesthesiol. 2024 Jun 26;74(5):844533. doi: 10.1016/j.bjane.2024.844533 (PMC11269778; doi:10.1016/j.bjane.2024.844533)

BJAN-D-23-00562_Supplementary Material

**Appendix A** Exclusion criteria.

| **Exclusion criteria** |
| --- |
| 1. Children with developmental delay |
| 2. Children with psychological/emotional disorders |
| 3. Children with language barrier |
| 4. Previous anesthetic or surgical experience |
| 5. Children whose eyes will be “closed” following surgery |
| 6. Children on sedative or psychoactive medication |
| 7. History of allergy to medications in our study |
| 8. Children with expected difficult intubation |
| 9. Children presenting for emergency surgery |
| 10. Family history or personal history of malignant hyperthermia/risk of MH |
| 11. Consent not obtained or withdrawal of consent |
| 12. Children who are violent during induction of anesthesia |
| 13. Cancellation of surgery |

Changes made to the protocol: The exclusion criteria “previous anesthetic or surgical experience” was removed for the high frequency of patients previously anesthetized at our centre and impossibility to recruit.

**Appendix C** Study flow and time-points for collecting data.


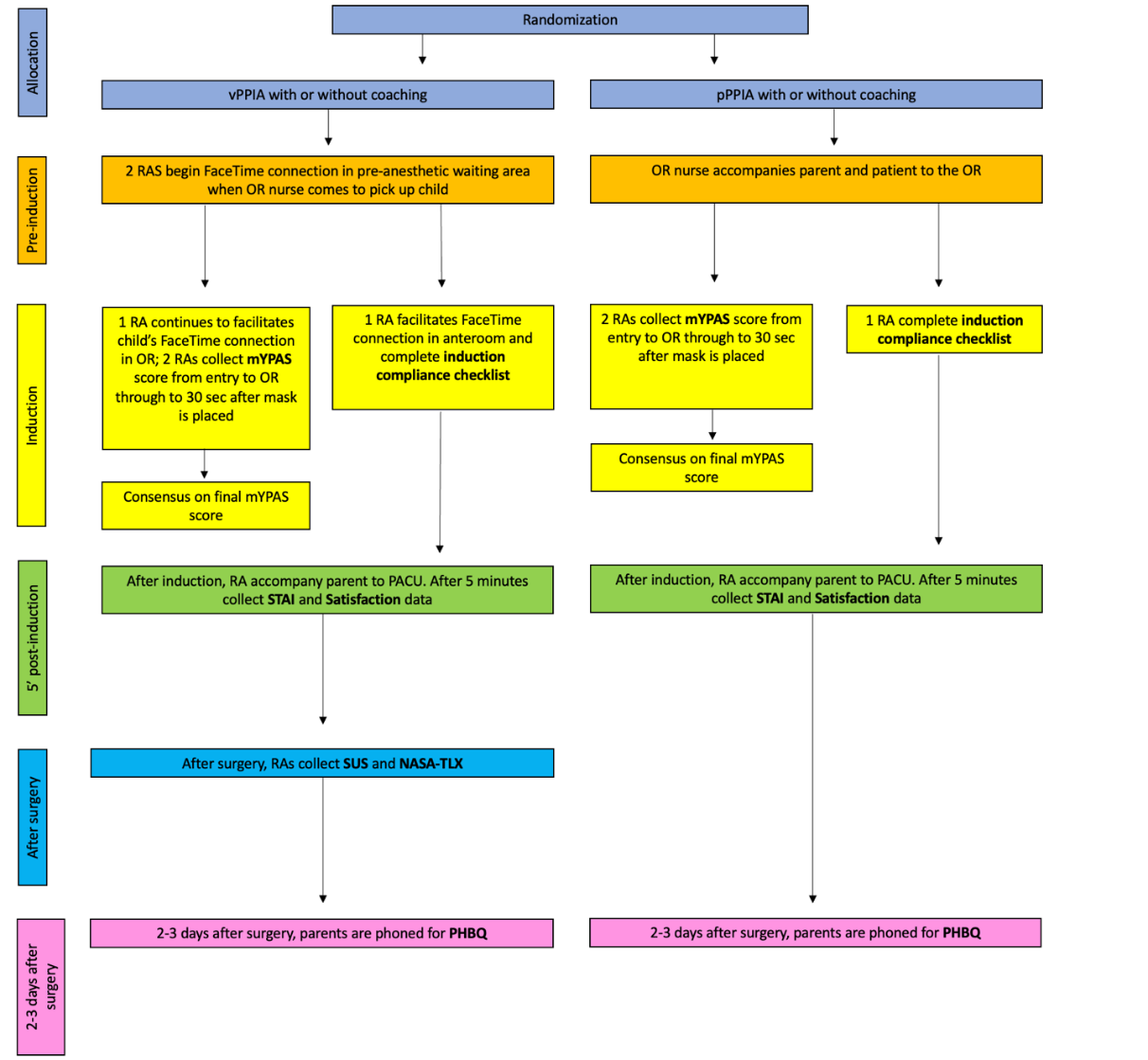


**Appendix D** NASA-TLX and SUS scores of anesthesiologists and induction nurses.


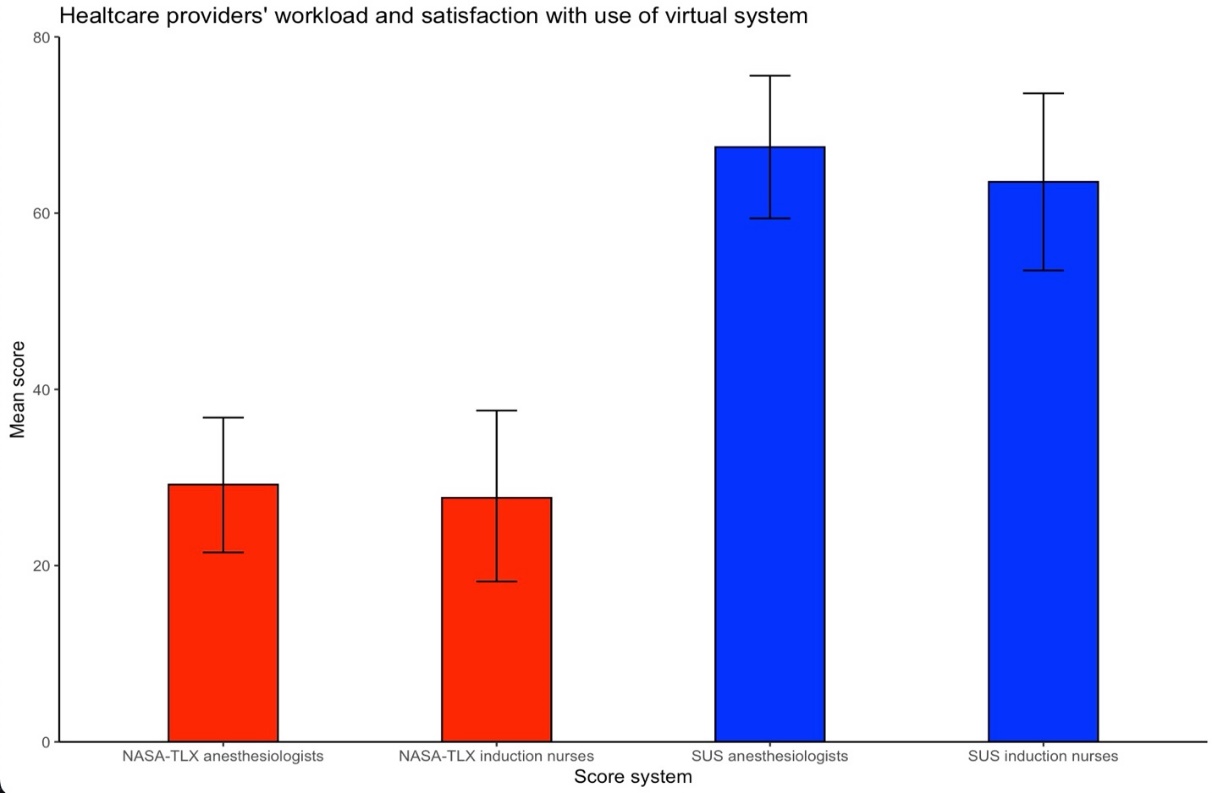

Supplement: Supplementary file 1 [file mmc1.docx]
