# Supplementary material for: Virtual parental presence with coaching for reducing preoperative anxiety in children: a feasibility and pilot randomized controlled trial
Source: Braz J Anesthesiol. 2024 Jun 26;74(5):844533. doi: 10.1016/j.bjane.2024.844533 (PMC11269778; doi:10.1016/j.bjane.2024.844533)

Parental Presence for Induction of anesthesia

SickKids® 2025

Getting ready for the OR

- What to expect in the operating room
- How you can help support your child
- What to expect during induction
- What happens after your child is asleep

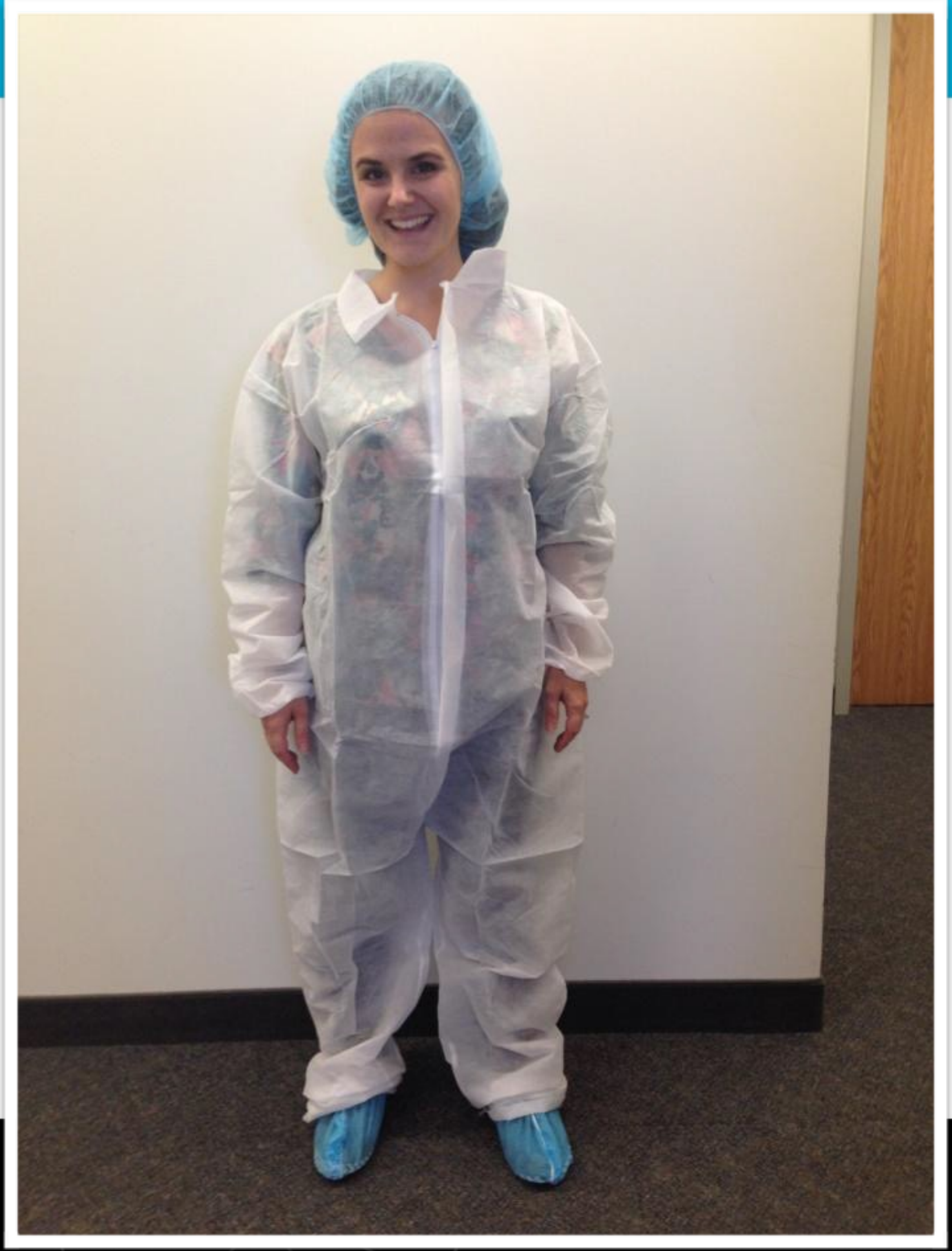

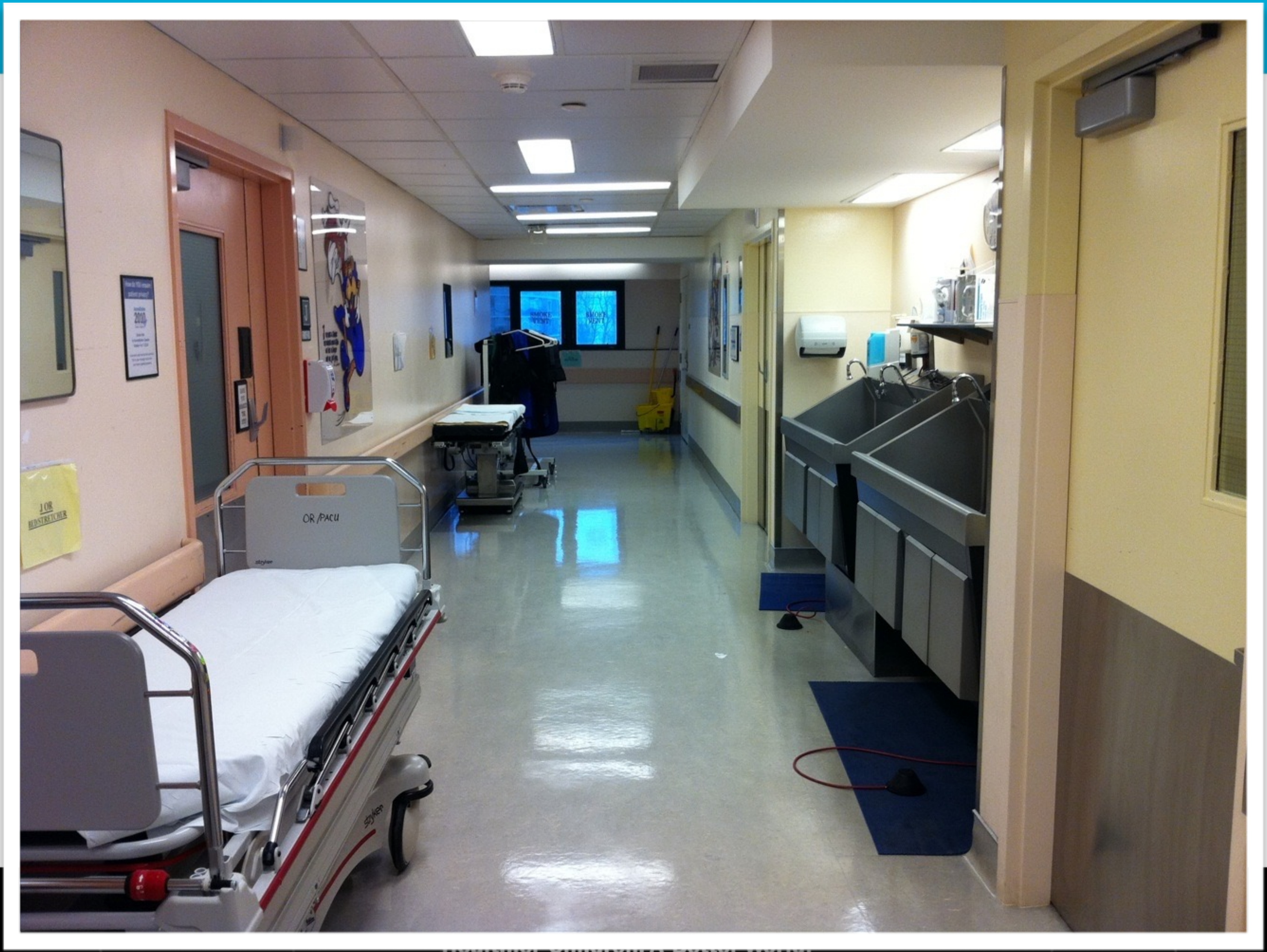
J OR
BEDSTRETCHER
OR/PACU

- Getting ready for the OR
    - What to expect in the operating room
    - How you can help support your child
    - What to expect during induction
    - What happens after your child is asleep

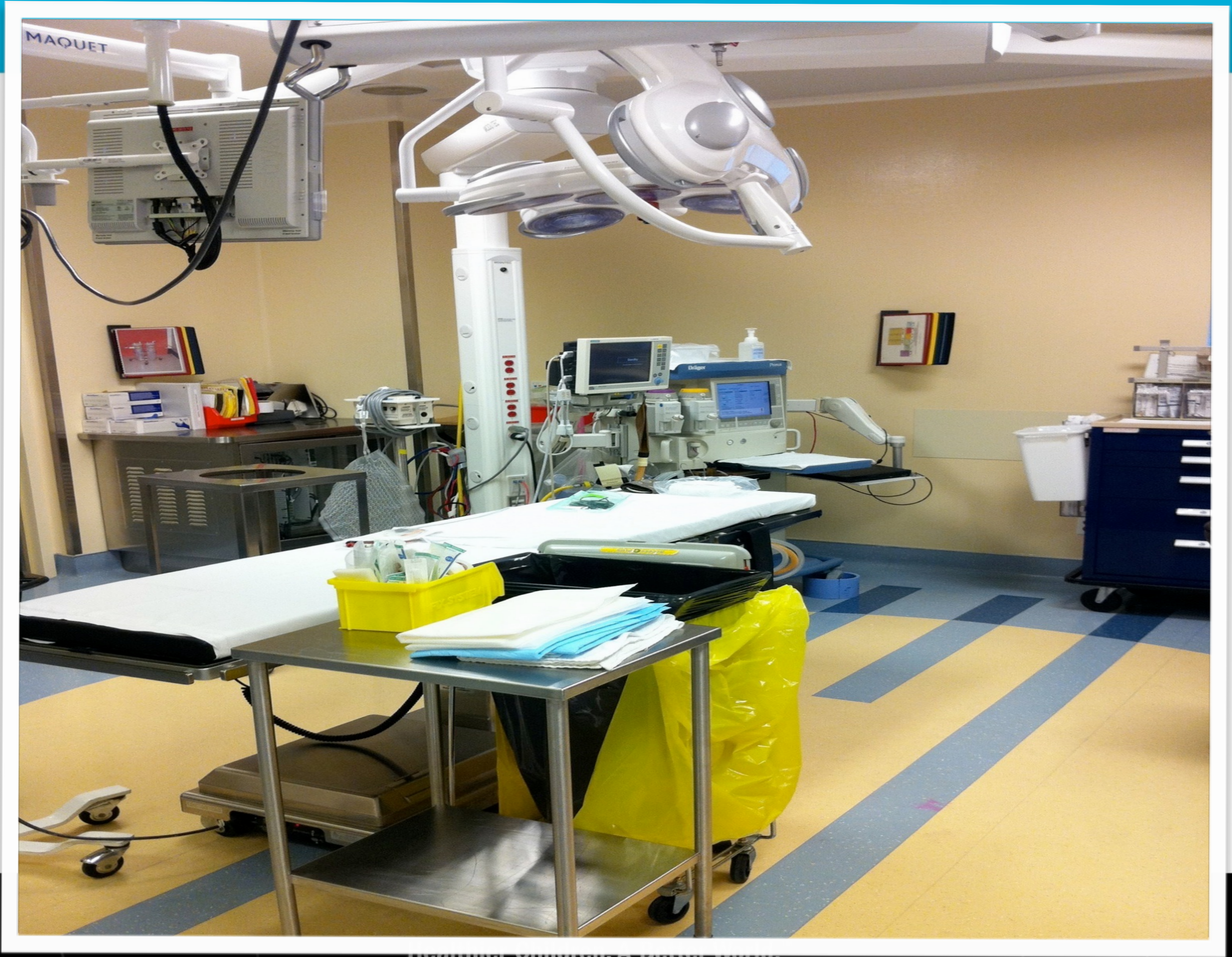
MAQUET

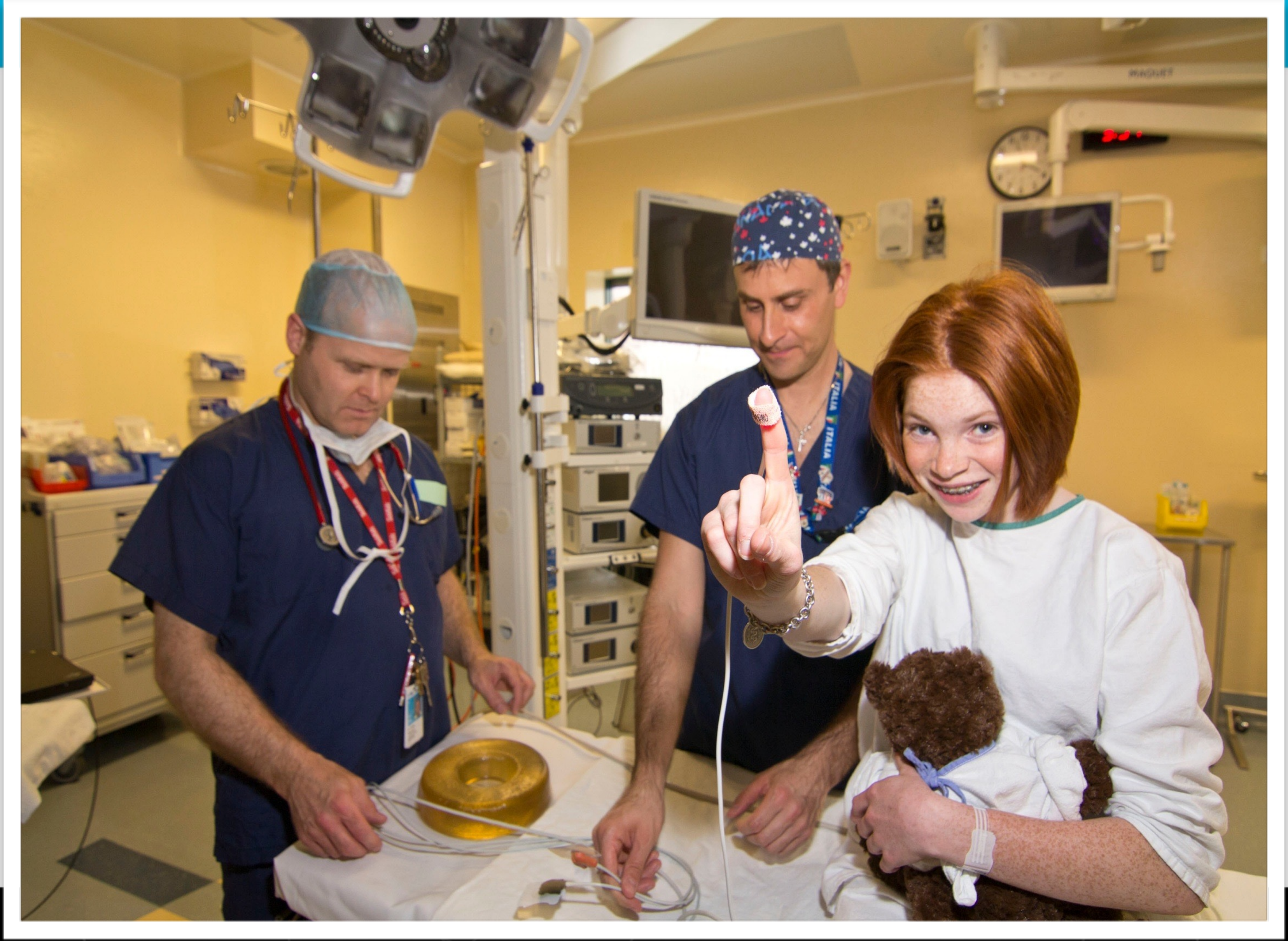

- Getting ready for the OR
- What to expect in the operating room
- How you can help support your child
- What to expect during induction
- What happens after your child is asleep

- **Do use** distraction. Talk with your child and make comments that steer their attention away from the medical procedure.
- **Avoid** focusing on your child's stress.
  - Ex: Tell stories about pets or family vacations, and avoid saying, I'm sorry, don't worry, it's OK.

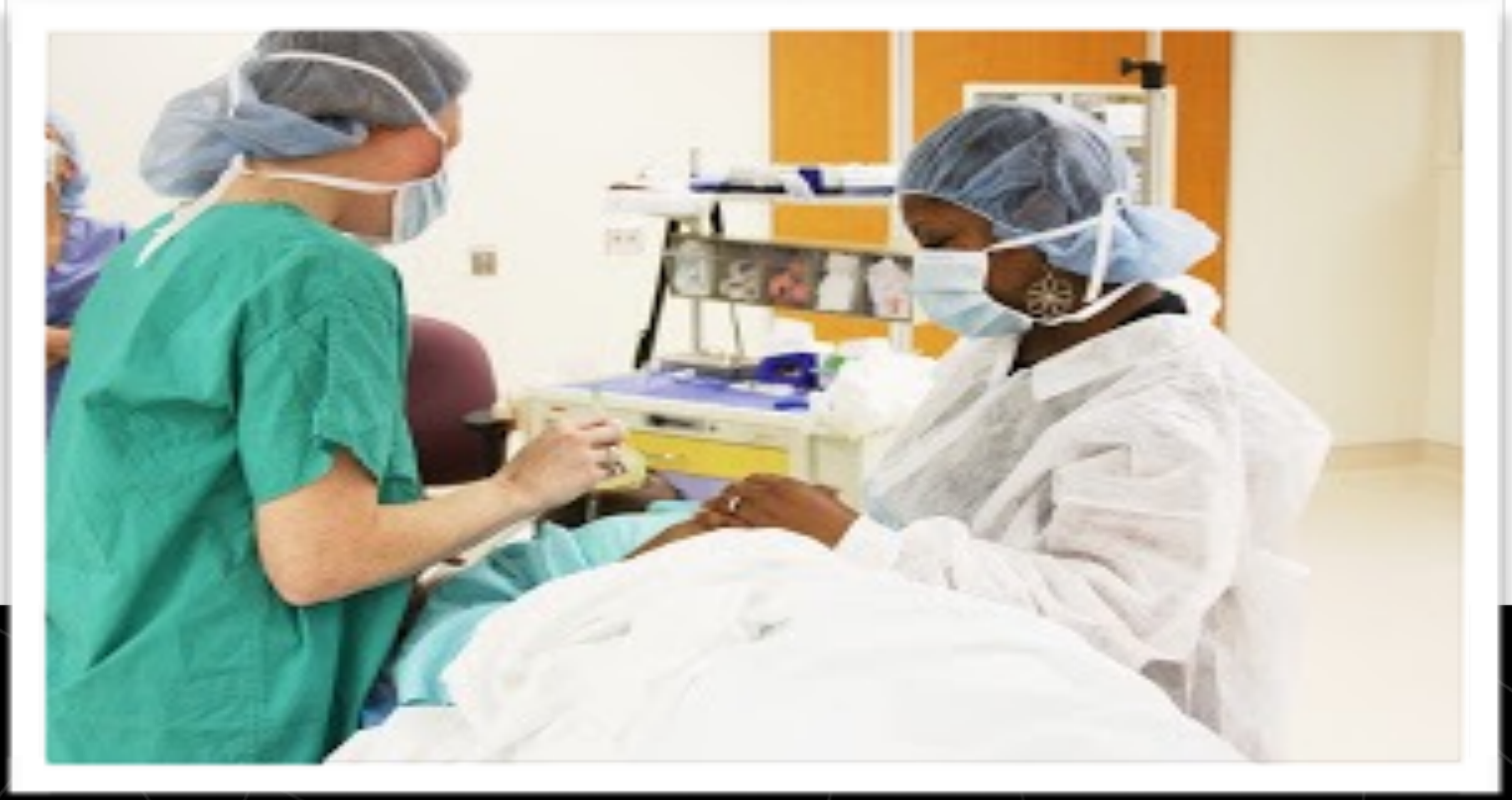

- **Do** give real choices: Offer choices that they are able to choose from.
- **Avoid** offering choices for things your child has no control over.
  - ex: "Would you rather I hold your hand or stroke your hair?" instead of, "Are you ready to breathe into the mask?"

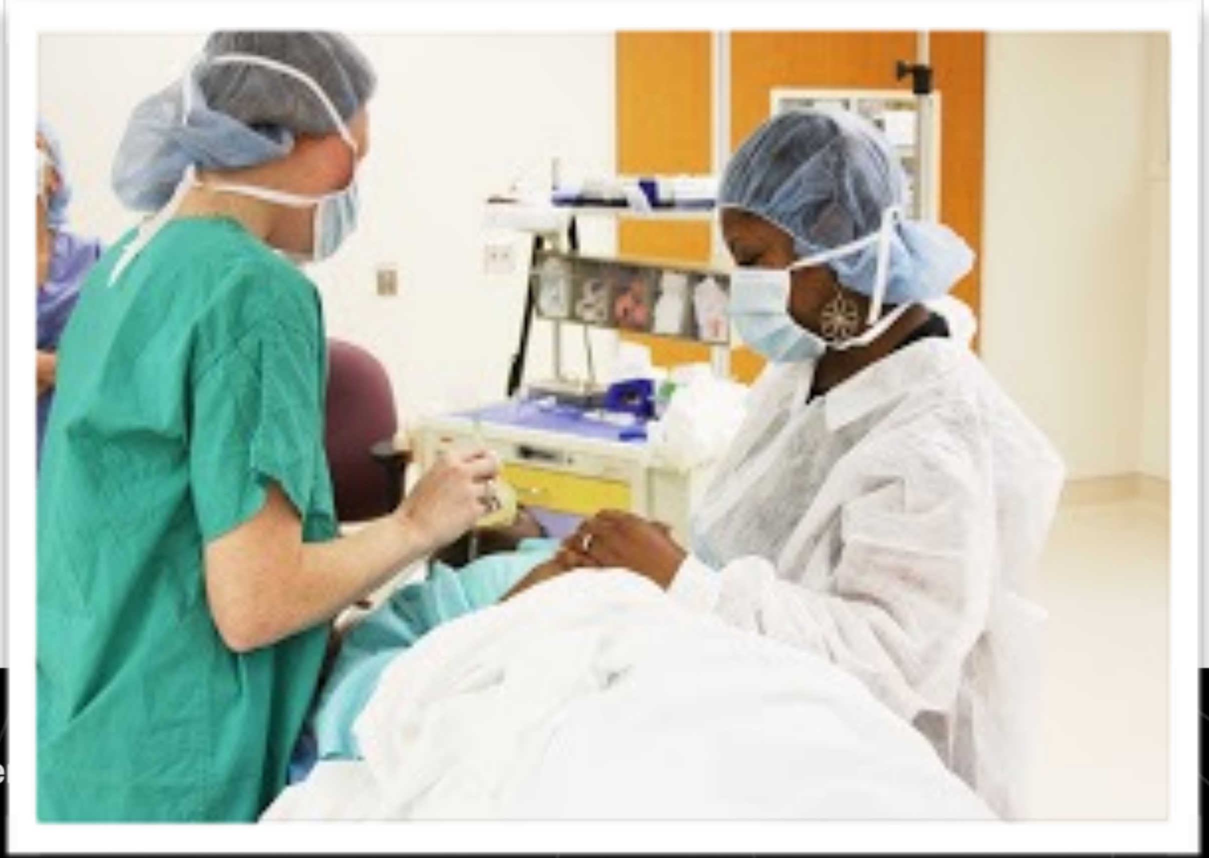

- **Do** make the medical equipment your child can see fun and positive.
- **Avoid** talking about equipment your child can't see.
  - ex: "Let's pretend you are breathing into an astronaut's mask and you are about to take off into outer-space."

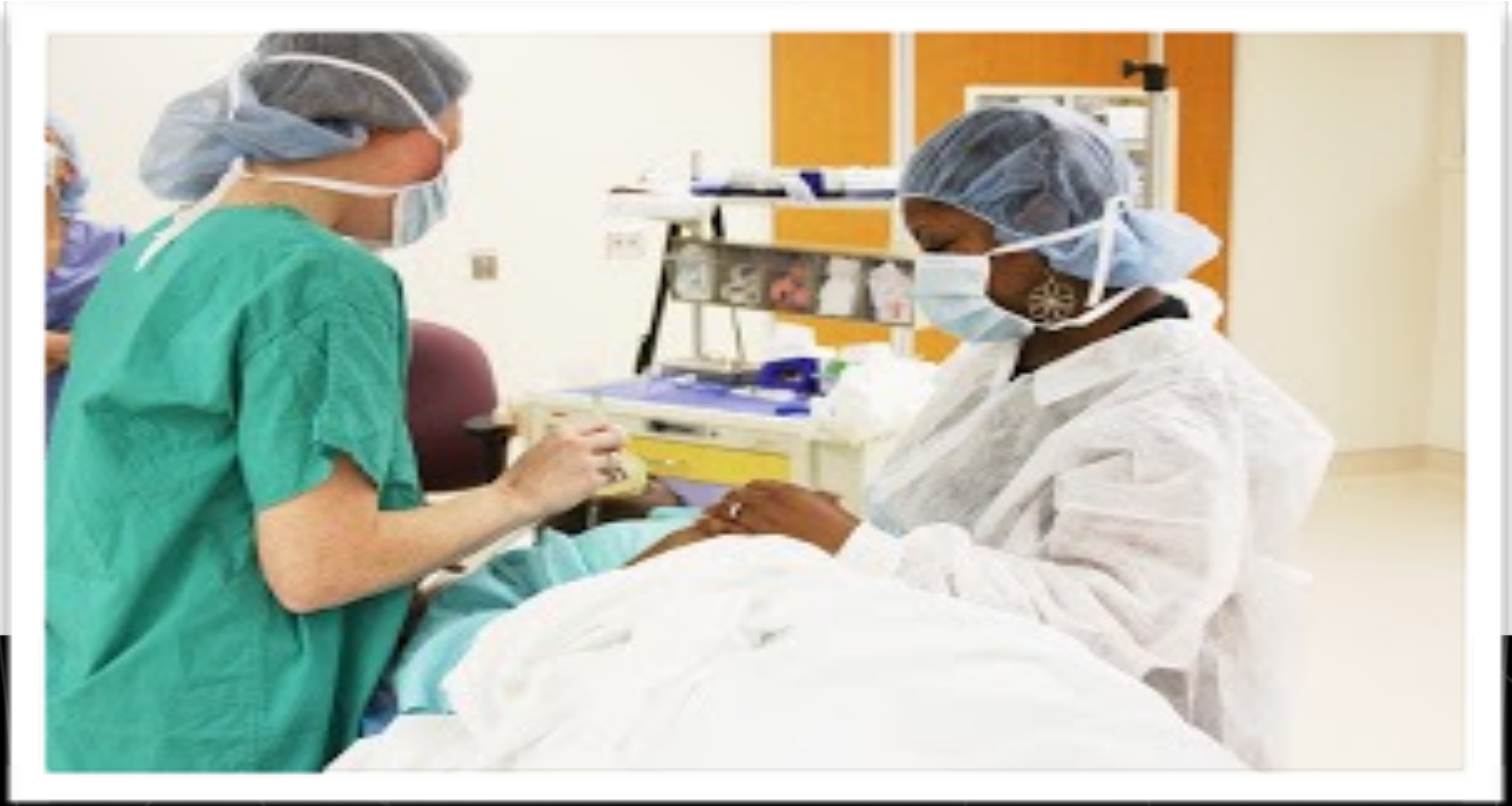

- **Do** use humour: Give your child something positive to focus on by being silly or telling jokes.
- **Avoid** giving too many technical details about the procedure or equipment.
- Note: Each child will respond differently to the suggestions given here. If you find your child getting more nervous, try a different strategy.

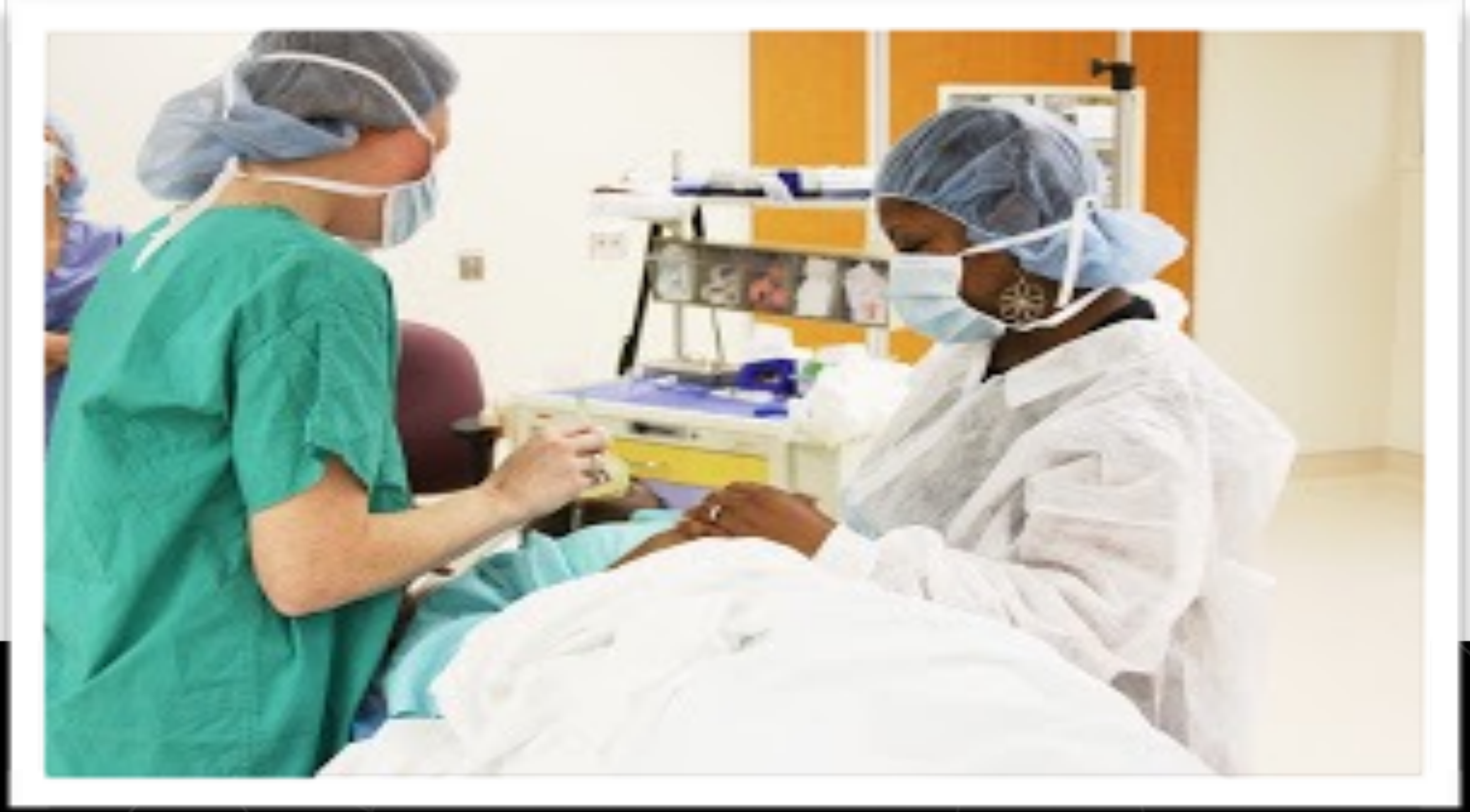

- Getting ready for the OR
- What to expect in the operating room
- How you can help support your child
- What to expect during induction
- What happens after your child is asleep

Excitement phase of anesthesia

Your child may experience any of the following:

- eyes rolling back or side to side
- wriggling, wrestling, or becoming rigid
- attempting to remove the mask

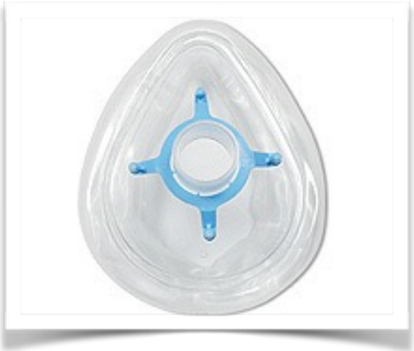

Getting Ready for the OR

- What to expect in the operating room
- How you can help support your child
- What to expect during induction
- What happens after your child is asleep

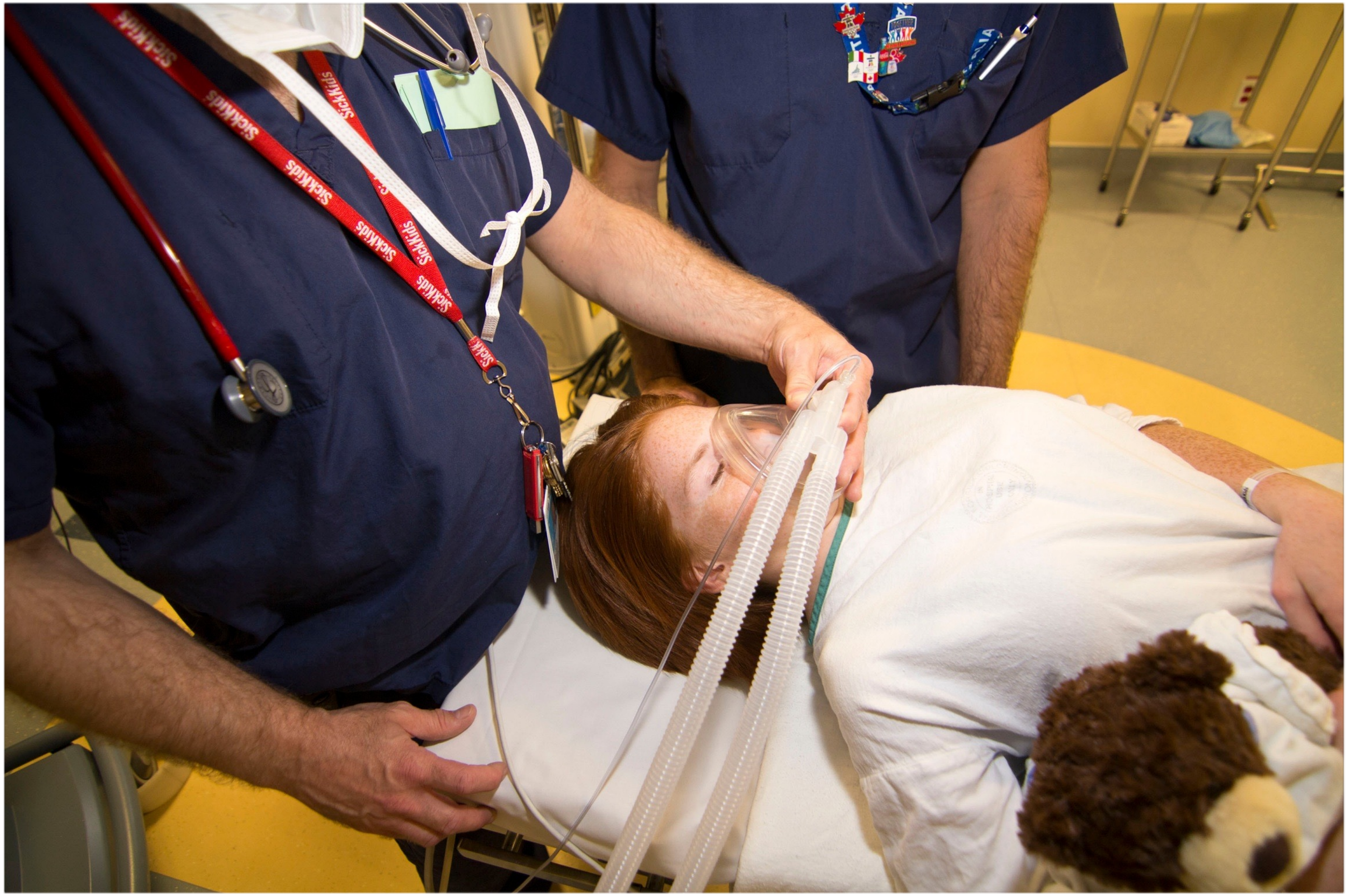
SickKids

The end

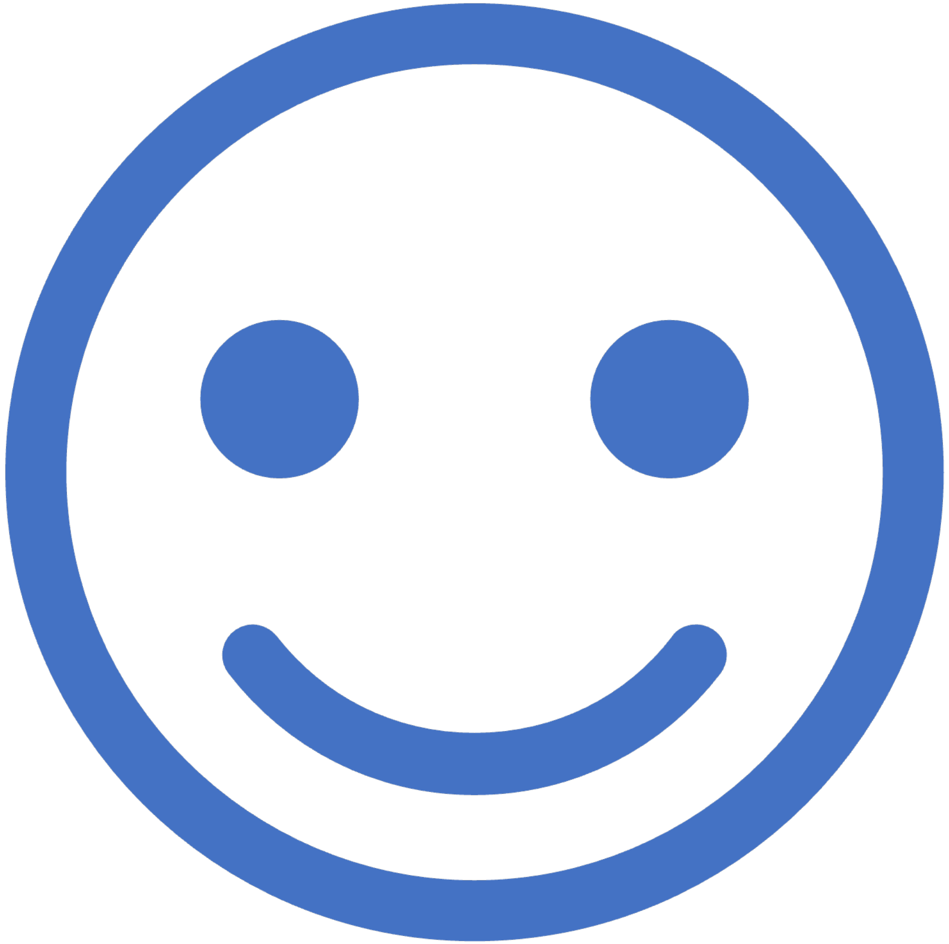

Supplement: Supplementary file 2 [file mmc2.pdf]
